# High accuracy tracking of ultrasonic motor based on PID operation of sliding surface plus inverse system compensation

**DOI:** 10.1038/s41598-022-10632-y

**Published:** 2022-04-26

**Authors:** Gangfeng Yan

**Affiliations:** 1 College of Electronic Information and Electrical Engineering, Chengdu University, ChengDu, 610106 Sichuan China; 2 Anhui Province Key Laboratory of Detection Technology and Energy Saving Devices, Anhui Polytechnic University, Wuhu, 241000 Anhui China; 3 Key Laboratory of Advanced Manufacturing Technology, Ministry of Education, Guizhou University, GuiYang, 550025 Guizhou China

**Keywords:** Electrical and electronic engineering, Mechanical engineering

## Abstract

Ultrasonic motor as a actuator of control system is widely used in the equipment driven for the precision manufacturing. In this brief, for the selection of the ultrasonic motor, an approximate time-domain mathematical model was established according to the physical mechanism of the ultrasonic motor. The parameters of the model were identified by using the least square method. Responses of the obtained model to the pulse width signal and the triangular wave signal are approximate consistent with those of the actual system respectively, which show the accuracy of the model. Then, the approach of PID operation of the sliding surface plus the inverse system compensation is proposed, the stability of the controlled system and the selection of the proposed approach parameters were discussed. The conventional PI control method with large gain and the proposed control approach were used to track the same signal. Then, the robustness of the proposed control method was tested, a 0.3 kg load was added to the system while keeping the two controller parameters and tracking signals unchanged, and the tracking effects of the two control methods were obtained. The results show that the proposed control approach has a superior performance compared to the conventional PI control approach.

## Introduction

With the development of industrial technology, there is an urgent need to develop micrometer positioning technology in the fields of precision manufacturing such as micromanipulators^1–3^, atomic force microscopes^4–7^, and ultra-precision machine tools^8–10^. The traditional electromagnetic motors are difficult to meet this demand because they are characterized by the high speed and low torque. Ultrasonic motors use piezoelectric ceramics as transducers, and utilize the inverse piezoelectric effect of piezoelectric ceramics to convert the electrical energy to the mechanical energy, can masterly utilize certain structural forms to transfer motions and motive forces. The most significant difference of the ultrasonic motor and the traditional electro-magnetic motors is no electromagnetic field and is not affected by electromagnetic fields. In addition, due to the compact structure of ultrasonic motors, the rotor inertia is small, fast in response to braking, the load can be directly driven, and has good controllability for positioning and speed, ultrasonic motor as system actuator are widely used in equipment drive and control with high performance for precision manufacturing. Ultrasonic motors play the role of the traditional electromagnetic motor, which is difficult to replace, but the high precision scale of ultrasonic motor gives rise to extra difficulty for establishing an accurate mathematical model of it as any tiny factors, nonlinear, interference and unknown, will sharply affect their characteristics. Therefore, ultrasonic motor have attracted more and more attention from researchers.

The inherent nonlinearities and vibrational behaviors in the ultrasonic motors are the most challenge for the modeling and control of the ultrasonic motors^11–15^. For repeated tracking tasks, iterative learning control can be used. Iterative learning control obtains the control input that can produce the desired output trajectory by repeatedly applying the information obtained from previous experiments to improve the quality of control^16,17^. For most non-repetitive tracking tasks, a series of feedback control schemes have been proposed for high accuracy tracking of ultrasonic motor, such as proportional-integral derivative control^18^, sliding mode control^19^, fuzzy decentralized control^20^ and H∞ control^21^, which schemes can achieve acceptable tracking accuracy and interference suppression performance, but due to the low gain margin and inherent sampling delay of the ultrasonic motor, the control performance could be reduced, this is especially true for high frequency tracking tasks. Among the various methods, sliding mode control is a kind of essential nonlinear control. Although the sliding mode control has good robustness to system perturbation and interference, its inherent chattering problem and the characteristics of the uncertain system to satisfy the matching condition are limited the application in engineering practice^22–25^.

In this paper, an approximate time-domain mathematical model is established for the selected ultrasonic motor system. Based on this model, a new approach of the PID operation for sliding surface plus inverse system feed-forward compensations designed to achieve the precision control, which has no switching action, thus avoiding the generation of chattering, so it has good practical value in the drive and control of precision instruments and equipment.

The following parts of this brief are organized as follows. First, aim at the selection of the ultrasonic motor, Sect. 2 of the brief introduces the process of establishing its mathematical model in detail. The PID operation of sliding surface approach plus inverse system feed-forward compensation is compared with the traditional PI control for tracking the same signal are introduced in Sect. 3 along with a stability analysis and discussion on parameter setting for the control algorithm proposed in this paper, some conclusions are summarized in Sect. 4.

## Experimental setup and dynamic modeling

### Experimental setup

**Figure 1:**
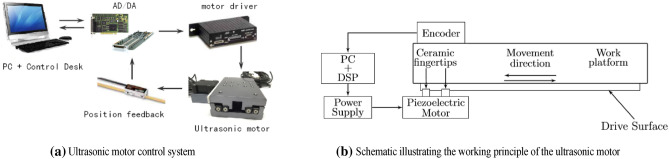
Ultrasonic motor control system.

In this brief, an ultrasonic motor is used as the plant, which has the full range of 115 mm. Its max velocity is 230 mm/s ^26^. The ultrasonic motor is equipped with low-voltage piezoelectric drives integrated into the system. The encoder is the Mercury 3000 made, its resolution is 20 nm^27^. The controller is implemented by using the MATLAB in a host computer containing a dSPACE DS1104 card. The DS1104 card is a powerful controller board for rapid control prototyping. The dSPACE Control Desk is used as the user interface, through this software, experiments are performed with parameter adjustments and measurements made in real time. The entire ultrasonic motor control system is shown in Fig. 1(a).

The working principle of the ultrasonic motor is to use the inverse piezoelectric effect of piezoelectric ceramics. The schematic diagram of the principle is shown in Fig. 1(b). After the driving voltage is input to the ultrasonic motor table of friction transmission, the piezoelectric ceramics in the piezoelectric motor will produce the inverse piezoelectric phenomenon, which leads to the longitudinal extension and the transverse bending deformation, and generates an ultrasonic standing wave in the narrow elliptical channel where the ceramic fingertip is located, and the ceramic fingertip squeezing the drive belt will produce a drive in the direction of movement as shown in Fig. 1(b). The drive circuit inside the piezoelectric motor controls the two ceramic fingertips to produce high-frequency alternating vibrations. The friction between the alternating ceramic fingertips and the drive surface drives the work platform fixed on the drive surface to act as linear movement in the direction shown in Fig. 1(b). When there is no driving voltage input, the pressure of the ceramic fingertips on the drive surface can maintain a holding torque on the working platform without movement.

### Dynamic modeling

An ultrasonic motor consists of a plat form that slides on rigid rails and, as such, friction plays a major role in the disturbance that effects the performance of the system. In order to establish the mathematical model of ultrasonic motor control system as accurately as possible, first, we need to briefly describe the types of frictional forces and their mathematical description in the ultrasonic motor systems^16^.

#### Type of friction force

##### Static friction force

At zero velocity, the static friction force opposes all motion as long as the force is smaller in magnitude than the maximum static friction force *f*_*s*_, and is usually described by through experiments, static friction force is discontinuous when the velocity crosses zero. Static friction force is described by

1 $$  F_{s}  = \left\{ {\begin{array}{*{20}l}    {u,} \hfill & {|u| < f_{s} } \hfill  \\    {{\text{ }}f_{s} {\text{sgn}}(u)\delta (\dot{x})} \hfill & {|u| \ge f_{s} } \hfill  \\   \end{array} } \right. $$

where *F*_*s*_ denotes the static friction force, *f*_*s*_ denotes the maximum static friction force, *u* is the applied force, $\dot{x}$ is the velocity of movement, ${\text{sgn}} (u)$ is a symbolic function, $\delta (u)$ is a unit pulse function.

##### Coulomb friction force

Coulomb friction is a type of mechanical damping in which energy is consumed via sliding friction. The friction generated by the relative motion of the two surfaces that press against each other always resists relative motion and is proportional to the normal force of contact.

Coulomb friction *f*_*c*_ is described by

2 $$ F_{c} = f_{c} {\text{sgn}} (\dot{x}) $$

where *f*_*c*_ is the normal force.

##### Viscous friction force

Viscous friction, is a resistance force that acts on an object in motion. This resistance acts against the motion of any object through another and also against motion of the object itself past stationary obstacles. Under well-lubricated conditions, the viscous friction force is approximately proportional to velocity. It satisfies the linear relationship given as

3 $$ F_{v} = f_{v} \dot{x} $$

where *f*_*v*_ is the coefficient of viscous friction.

##### Drag friction force

Drag friction is the friction force between a solid object and a liquid or a gas. It is proportional to the square of velocity, drag friction is described by

4 $$ F_{d} = f_{d} \dot{x}|\dot{x}| $$

where *f*_*d*_ is the drag coefficient.

Classical friction models have different combinations of static, coulomb, viscous and drag friction as their basic components.

#### System modeling

**Figure 2:**
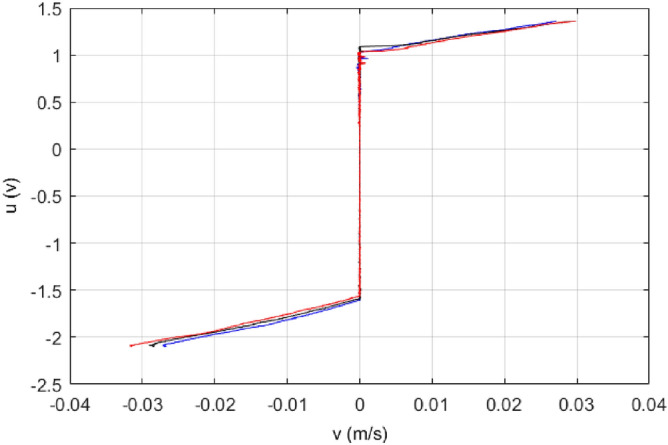
Experimental results of input force u w.r.t. velocity v.

A number of experimental tests are conducted, and the results of three tests are shown in Fig. 2.

During the experiment, a slow triangular input acts on the ultrasonic motor to generate low-speed motion with low acceleration, in this way, the input force acting is only to overcome the friction of the ultrasonic motor, therefore, the force-speed relationship in Fig. 2 can be obtained. When modeling the ultrasonic motor in performance analysis, from the Fig. 2, it can be seen that static friction force, Coulomb friction force and viscous friction force need to be considered. The speed of the ultrasonic motor is very small, and the coefficient of friction of air against solids is also very small, so in this case the drag friction force can be ignored. Note that the forward and backward friction coefficients of the ultrasonic motor are different. Considering the static friction force, Coulomb friction force and viscous friction force, the dynamics of the ultrasonic motor can be expressed by the following second-order differential equation with quality normalization by using Newton's second law

5 $$ \ddot{x} = - \frac{{k_{1} }}{m}\dot{x} - \frac{{k_{2} }}{m}{\text{sgn}} (\dot{x}) - \frac{1}{m}F_{s} + \frac{{k_{3} }}{m}u $$

where ${\mathrm{k}}_{1}$ *k*_1_ is the coefficient of viscous friction, *k*_2_ is the normal force, m denotes the rotor mass of ultrasonic motor, in order to facilitate writing, let

6 $$ a_{1}  = \frac{{k_{1} }}{m} = \left\{ {\begin{array}{*{20}l}    {a_{{1p}} } \hfill & {\dot{x} > 0} \hfill  \\    {a_{{1n}} } \hfill & {\dot{x} < 0} \hfill  \\   \end{array} } \right. $$

7 $$ a_{2}  = \frac{{k_{2} }}{m} = \left\{ {\begin{array}{*{20}l}    {a_{{2p}} {\text{ }}} \hfill & {\dot{x} > 0} \hfill  \\    {a_{{2n}} } \hfill & {{\text{ }}\dot{x} < 0} \hfill  \\   \end{array} } \right. $$

8 $$ a{}_{3} = \frac{{k_{3} }}{m} $$

**Figure 3:**
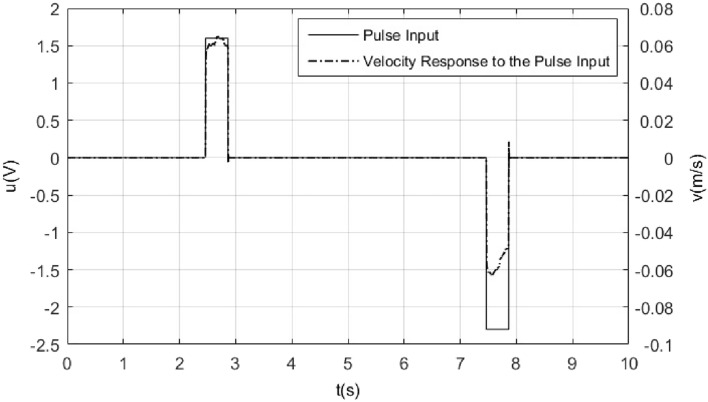
Pulse input of 0.4 s duration and with − 2.3 V, 1.6 V of amplitude and its velocity response.

*k*_3_ represents constants of the voltage to force conversion, and *a*_3_ = 6* N*/(V.kg), which is provided in the ultrasonic motor PLS8-115 product documentation^26^.In order to obtain the values of the remaining parameters in model (5), the system will be subjected to pulse inputs in order minimize the influence of static friction on the system. So, some pulse responses experiments are needed to make firstly. By using a pulse input of 0.4 s duration and with 1.6 V, − 2.3 V of amplitude, the velocity response to the pulse inputs is shown in Fig. 3.

From this experiment results, it is obtained that

9 $$ \left\{ {\begin{array}{*{20}c} {0.05562a_{1n} + a_{2n} - 6 \times 2.3 = 0} \\ { - 0.06222a_{1n} - a_{2n} + 6 \times 1.6 = 0} \\ \end{array} } \right. $$

Following a similar method, using a pulse of 0.4 s duration and amplitudes of − 1.8 V, 1.3 V, − 2 V, 1.5 V, − 2.1 V, 1.7 V, − 2.5 V, 2 V respectively, it is obtained that

10 $$ \left[ {\begin{array}{*{20}c} \begin{gathered} 0.03393 \hfill \\ 0.04622 \hfill \\ 0.04991 \hfill \\ 0.07120 \hfill \\ 0 \hfill \\ 0 \hfill \\ 0 \hfill \\ 0 \hfill \\ \end{gathered} & \begin{gathered} 0 \hfill \\ 0 \hfill \\ 0 \hfill \\ 0 \hfill \\ - 0.04465 \hfill \\ - 0.05742 \hfill \\ - 0.06863 \hfill \\ - 0.08519 \hfill \\ \end{gathered} & \begin{gathered} 1 \hfill \\ 1 \hfill \\ 1 \hfill \\ 1 \hfill \\ 0 \hfill \\ 0 \hfill \\ 0 \hfill \\ 0 \hfill \\ \end{gathered} & \begin{gathered} 0 \hfill \\ 0 \hfill \\ 0 \hfill \\ 0 \hfill \\ - 1 \hfill \\ - 1 \hfill \\ - 1 \hfill \\ - 1 \hfill \\ \end{gathered} \\ \end{array} } \right] \bullet \left[ \begin{gathered} a_{1n} \hfill \\ a_{1p} \hfill \\ a_{2n} \hfill \\ a_{2p} \hfill \\ \end{gathered} \right] = \left[ \begin{gathered} 10.8 \hfill \\ 12.0 \hfill \\ 12.6 \hfill \\ 15.0 \hfill \\ - 7.8 \hfill \\ - 9.0 \hfill \\ - 10.2 \hfill \\ - 12.0 \hfill \\ \end{gathered} \right] $$

Let the above formula correspond to ***X***·***A*** = *Y*, We use ***e*** to denote the difference between $\mathrm{Y}$ ***Y*** and ***X***·***A***, define $E = e^{T} e$, Using the least-squares method, let

11 $$ \frac{\partial E}{{\partial A}} = 0 $$

It is obtained that

12 $$ A = (X^{T} X)^{ - 1} X^{T} Y $$

*a*_*1n*_, *a*_*1p*_, *a*_*2n*_, *a*_*2p*_ can be obtained, by solving the Eq. (12), as

13 $$ \left[ \begin{gathered} a_{1n} \hfill \\ a_{1p} \hfill \\ a_{2n} \hfill \\ a_{2p} \hfill \\ \end{gathered} \right] = \left[ \begin{gathered} {117}{\text{.1441}} \hfill \\ {104}{\text{.0154}} \hfill \\ {6}{\text{.8216}} \hfill \\ {3}{\text{.1023}} \hfill \\ \end{gathered} \right] $$

**Figure 4:**
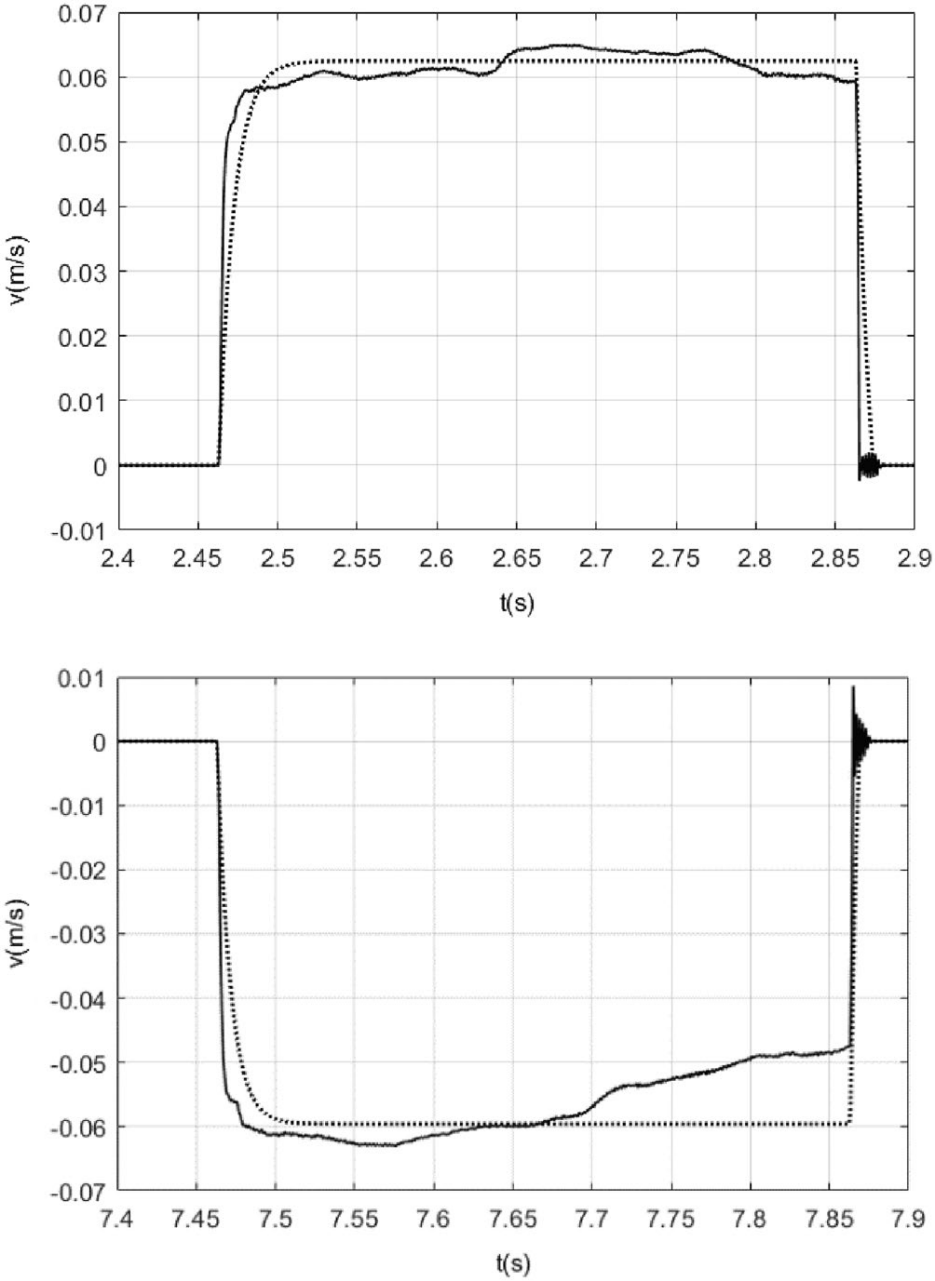
Contrasting curves for the response of the model (5) while ignoring static friction and the response of the actual system under the pulse input of 0.4 s duration and with1.6 V, − 2.3 V of amplitude.

Using model (5), while ignoring static friction, contrasting curves for velocities are obtained as a response to the pulses of amplitude 1.6 V and − 2.3 V, as shown in Fig. 4. In Fig. 4, the dotted line shows the response of the model while the solid line shows the response of the actual system.

**Figure 5:**
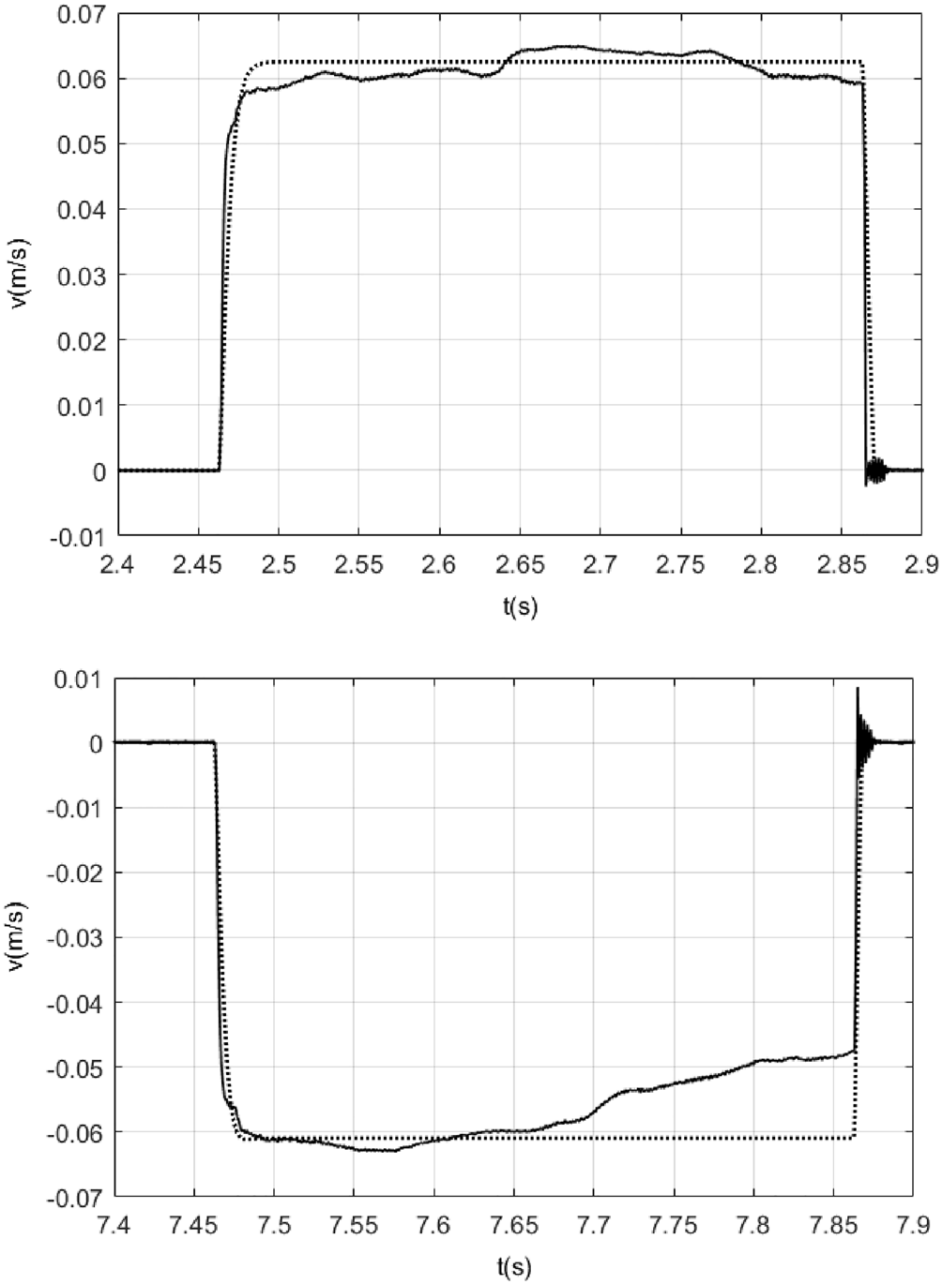
Contrasting curves for the response of the model with 0.0035 s delay of viscous friction for viscous friction in model (5) while ignoring static friction and the response of the actual system under the pulse input of 0.4 s duration and with 1.6 V, − 2.3 V of amplitude.

From Fig. 4, it was observed that the viscous friction force in model (5) has a certain delay, by adding the effect of viscous friction delay, repeated testing, when viscous friction delay is 0.0035 s, we can get the contrasting curves for the response of the model with this delay time for viscous friction in model (5) and the response of the actual system as shown in Fig. 5.

Static friction is difficult to accurately simulate as the system speed is zero at this time, so the model of the ultrasonic motor can be obtained for the performance analysis as follow

14 $$ \ddot{x}(t) = - a_{1} \dot{x}(t - 0.0035) - a_{2} {\text{sgn}} (\dot{x}) + 6u(t) $$

**Figure 6:**
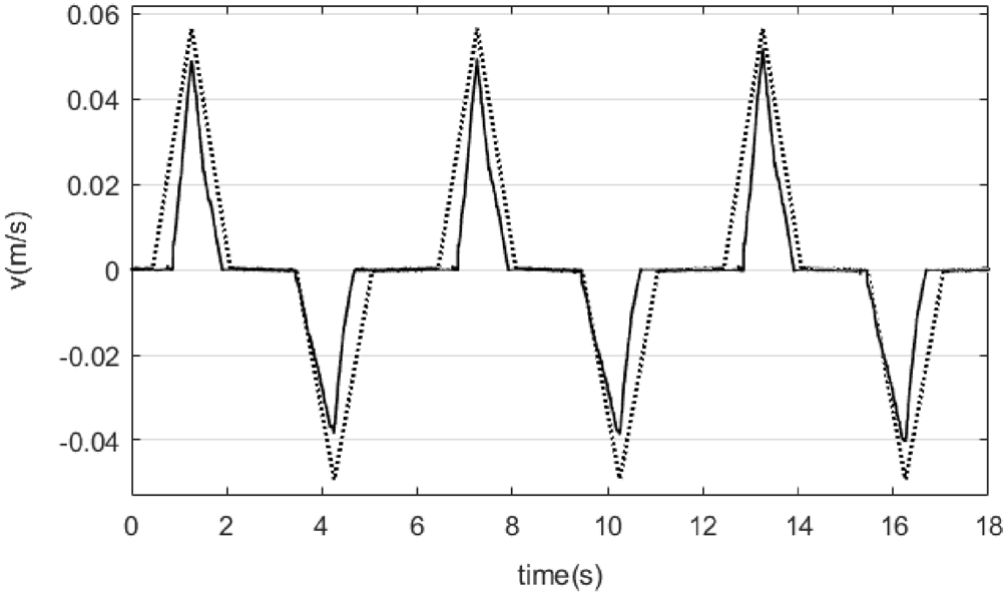
Contrasting curve for the velocity as a response to triangular function input with amplitude 1.5 V, − 2.1 V and a period of 6 s.

Using this model, contrasting curve for the velocity as a response to triangular function input with amplitude 1.5 V, − 2.1 V and a period of 6 s is shown in Fig. 6. The dotted line shows the results obtained by the model, and the solid line are the results of the actual system. It can be seen from the results that the simulation results are basically consistent with the actual system results, so the model can be used to design the control system.

## Control method design and testing

**Figure 7:**
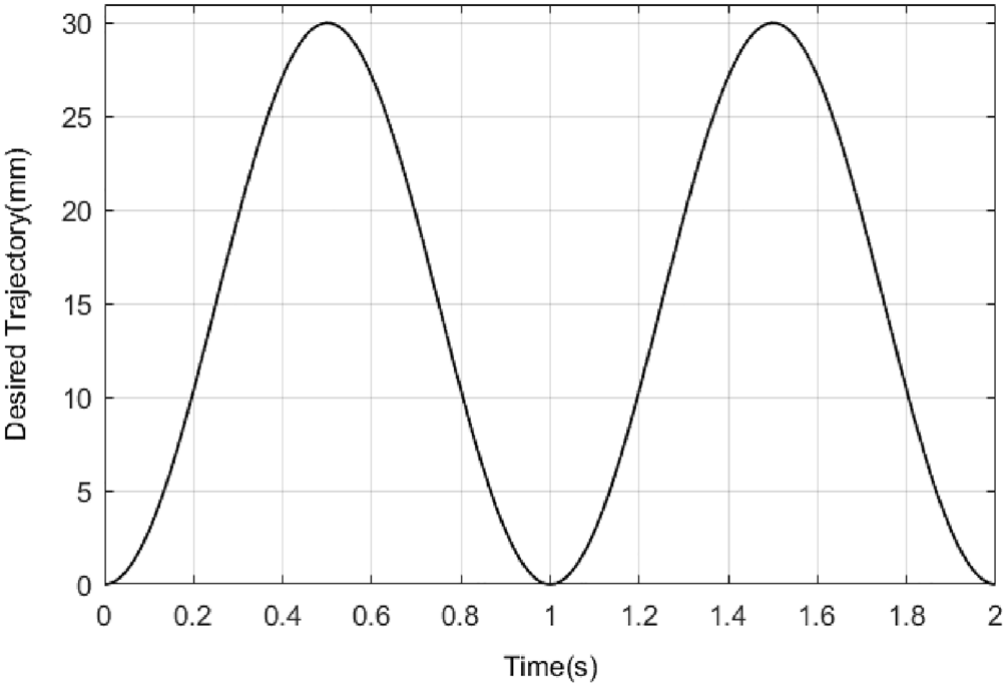
Desired tracking signal.

First, the position signal need to be tracked is shown in Fig. 7.

According to the system modeling results, the PID operation of sliding surface approach plus inverse system feed-forward compensation is designed as the control scheme for the ultrasonic motor control system, the sliding surface is defined as

15 $$ s(t) = e(t) + 5\dot{e}(t) $$

where *e*(*t*) = *x*_d_(*t*) − *x*(*t*) is the tracking error, ${\mathrm{x}}_{\mathrm{d}}(\mathrm{t})$ *x*_d_(*t*) denotes desired track trajectories, $\mathrm{x}(\mathrm{t})$ *x*(*t*) is the actual position signal.

The controller is designed as follow

16 $$ u(t) = \hat{u}(t) + \frac{1}{5}\dot{e}(t) + k_{P} s(t) + k_{I} \int_{0}^{t} {s(\tau )} d\tau + k_{D} \dot{s}(t) $$

where ${\mathrm{k}}_{\mathrm{P}}$ *k*_*P*_, *k*_*I*_, *k*_*D*_ are constants, according to the tracking error to determine. Note that, since the system interference is unavoidable, the output of $\dot{e}(t)$ needs to be increased by the filter according to the nature of the tracking signal, and then applied it for controller, a first-order low-pass filter with a time constant of 0.1 is to filter the signal $\dot{\mathrm{e}}\left(\mathrm{t}\right)$ $\dot{e}(t)$ in here.$\widehat{\mathrm{u}}\left(\mathrm{t}\right)$ $\hat{u}(t)$ is a feed-forward compensation control signal determined by the inverse system of the model (14)

17 $$ \hat{u}(t) = \frac{{ - \ddot{x}_{d} (t) + a_{1} \dot{x}_{d} (t - 0.0035) + a_{2} {\text{sgn}} (\dot{x}_{d} )}}{6} $$

**Figure 8:**
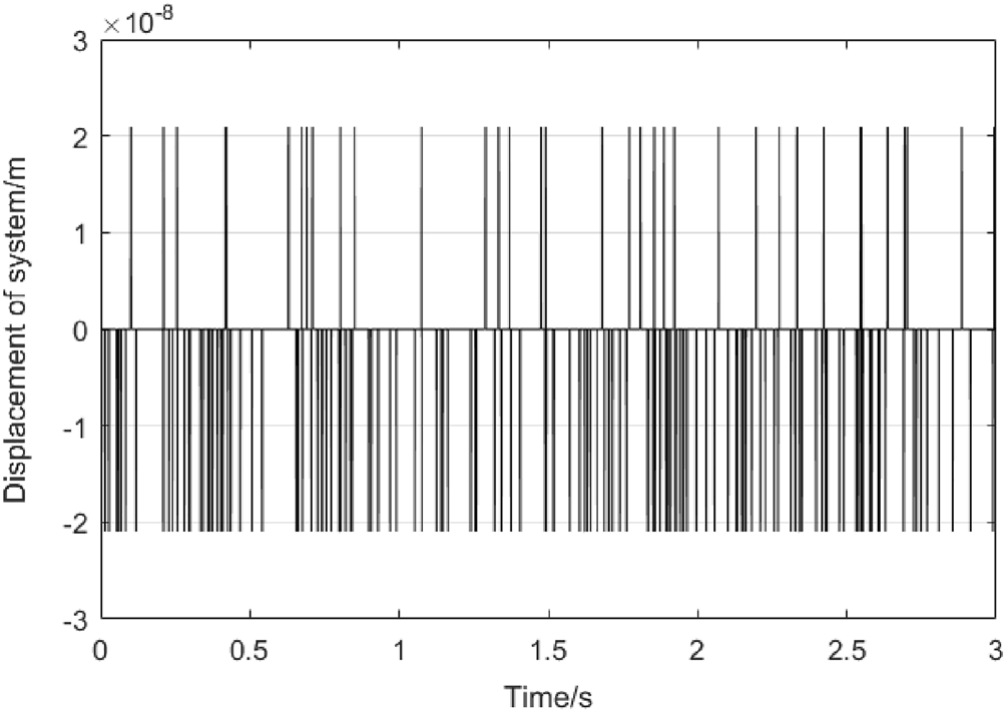
Encoder output without signal input to system.

Consider the actual system can be described as

18 $$ \ddot{x}(t) = f(x(t),\dot{x}(t)) + d(t) + u(t) $$

where *d*(*t*) is the system interference, the value in the ultrasonic motor control system is very small, which can be seen by the output of the encoder when the ultrasonic motor control system does not have input signal, which is shown in Fig. 8

The relationship between the partial model and the actual model is given by

19 $$ \tilde{f}(x(t),\dot{x}(t)) = f(x(t),\dot{x}(t)){ - }\hat{f}(x(t),\dot{x}(t)) $$

where $$\stackrel{\sim }{\mathrm{f}}\left(\mathrm{x}(\mathrm{t}),\dot{\mathrm{x}}(\mathrm{t})\right)$$$$\tilde{f}(x(t),\dot{x}(t))$$ is the modeling error, $$\widehat{\mathrm{f}}\left(\mathrm{x}(\mathrm{t}),\dot{\mathrm{x}}(\mathrm{t})\right)$$$$\hat{f}(x(t),\dot{x}(t)$$ is the modeling result, as will be described below, a controller (16) is selected for the control system (18), when the parameters in the controller are designed appropriately, and the control system (18) is stable. We choose the positive definite Lyapunov function as follow. For the sake of convenience, the following discuss will omit the time variable *t*.

20 $$ V = \frac{1}{2}s^{2} $$

Then

21 $$ \begin{gathered} \dot{V} = s\dot{s} \\ = 5s\left( { - k_{P} s - k_{I} \int_{0}^{t} {s(\tau )} d\tau - k_{D} \dot{s} - \tilde{f}(x_{d} ,\dot{x}_{d} ) - (f(x,\dot{x}) - f(x_{d} ,\dot{x}_{d} )) - d} \right) \\ \end{gathered} $$

where $$\tilde{f}(x_{d} ,\dot{x}_{d} )$$ is a constant and marked it as $${\stackrel{\sim }{\mathrm{f}}}_{\mathrm{dC}}$$ $$\tilde{f}_{dc}$$, Considering that is $$f(x,\dot{x})$$ bounded in a limited interval for the actual physical system, then it is assumed that $$\left|\mathrm{f}\left(\mathrm{x},\dot{\mathrm{x}}\right)-\mathrm{f}\left({\mathrm{x}}_{\mathrm{d}},{\dot{\mathrm{x}}}_{\mathrm{d}}\right)\right|<{\mathrm{K}}_{\mathrm{M}}|\mathrm{e}|$$$$||f(x,\dot{x}) - f(x_{d} ,\dot{x}_{d} )|| < K_{M} ||e||$$, where $${\mathrm{K}}_{\mathrm{M}}$$ *K*_*M*_ is a positive constant, The disturbance *d* is very small and assumed to be bounded as $$|\mathrm{d}|<\mathrm{D}$$||*d*||< *D*, therefore, reasonable choice for $${\mathrm{k}}_{\mathrm{P}}$$ *k*_*P*_, *k*_*I*_, *k*_*D*_, we can make the following relation is met

22 $$ k_{P} {|}|s|| + k_{I} \int_{0}^{t} {s(\tau )} d\tau + k_{D} \dot{s} > - (\tilde{f}_{dc} + K_{M} ||e|| + D) $$

Therefore, the time derivative of Lyapunov function (21) is negative definite. Further, according to the invariant set theorem, the system (18) with control approach (16) is the asymptotically stable.

In the compensation part of the control algorithm, the parameter ${\mathrm{k}}_{\mathrm{P}}$ *k*_*P*_ should select a larger value, and the parameters ${\mathrm{k}}_{\mathrm{I}}$ *k*_*I*_, and *k*_*D*_ should choose smaller values, which can shorten the time of the system reaching the sliding surface and reduce the speed of entering the sliding surface, which is beneficial to improve the control performance of the system, at the same time, it is easier to satisfy condition (22).

As a comparison, PI control is used to test the tracking performance of the ultrasonic motor under the same conditions. The parameters of PI control are proportional coefficient is 29,000 and integral coefficient is1900, we adjust and select the parameters of PI control based on the tracking error is minimal under the condition of not leading to oscillatory output.

**Figure 9:**
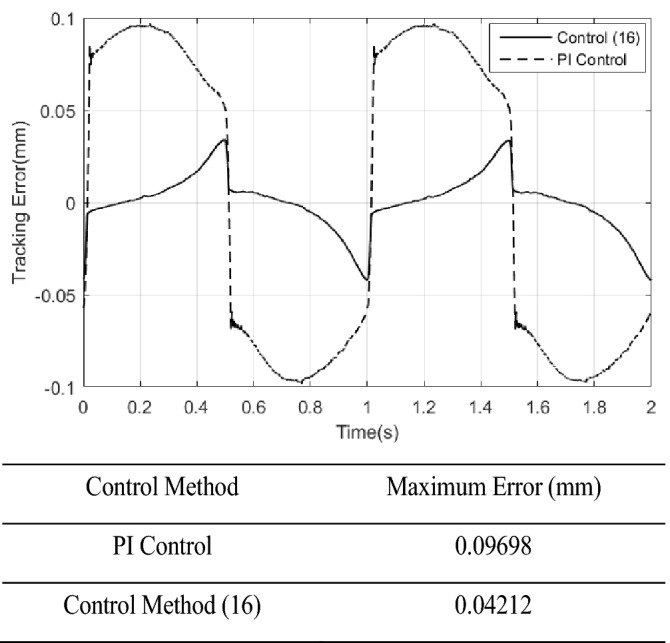
Tracking error of PI controller and control method (16).

**Figure 10:**
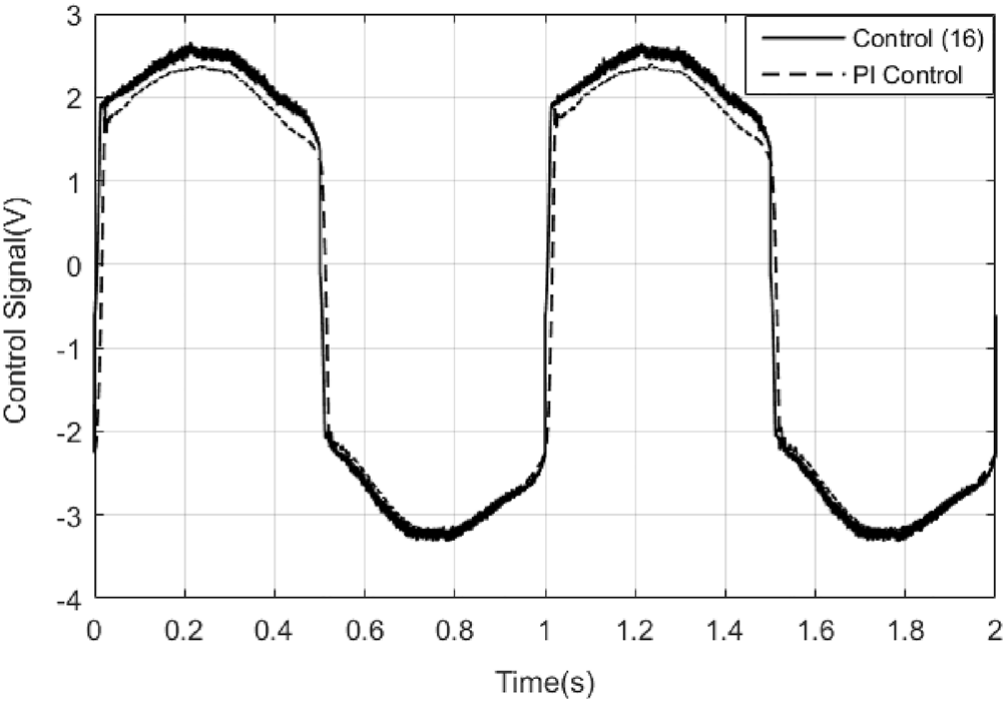
Comparison of the control signals of PI controller and control method (16).

**Figure 11:**
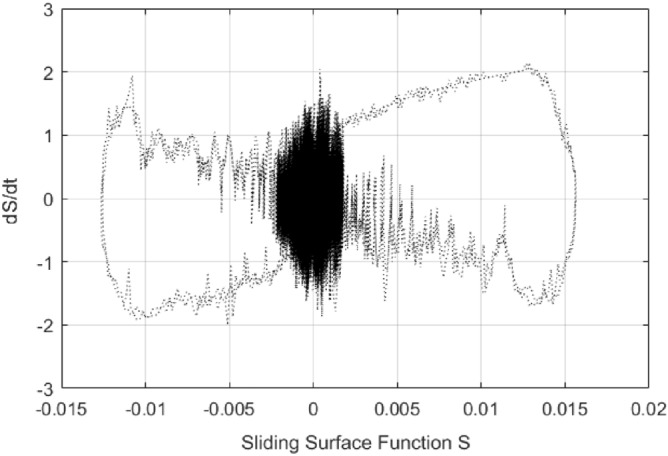
$s$ versus $\dot{s}$ in the phase plane.

According to the PID operation of sliding surface approach with inverse system compensation approach (16) given here, after repeated adjustment, the control parameters are ${\mathrm{k}}_{\mathrm{P}}$ *k*_*P*_ = 890,${\mathrm{k}}_{\mathrm{P}}=890$ ${\mathrm{k}}_{\mathrm{P}}$ *k*_*I*_ = 0.8, ${\mathrm{k}}_{\mathrm{P}}$ *k*_*D*_ = 2, the tracking error results of both PI control and control method (16) are shown in Fig. 9. It can be seen that the tracking error of the control method (16) is smaller in magnitude than that of the PI control. But when the PI control has the largest error, the control algorithm (16) has the minimum error, which result is due to the differential effect in the control algorithm (16) is too strong. The control signals of the control method (16) and PI control are shown in Fig. 10. The sliding surface function $s$ and its derivative $\dot{s}$ are shown in Fig. 11. It can be seen that $s$ and $\dot{s}$ are well convergent in the phase plane.

**Figure 12:**
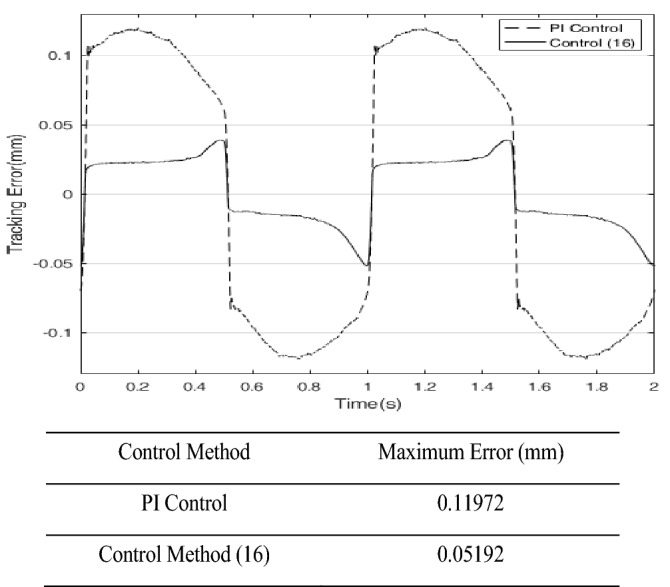
Tracking error of PI controller and control method (16) with 0.3 kg load.

**Figure 13:**
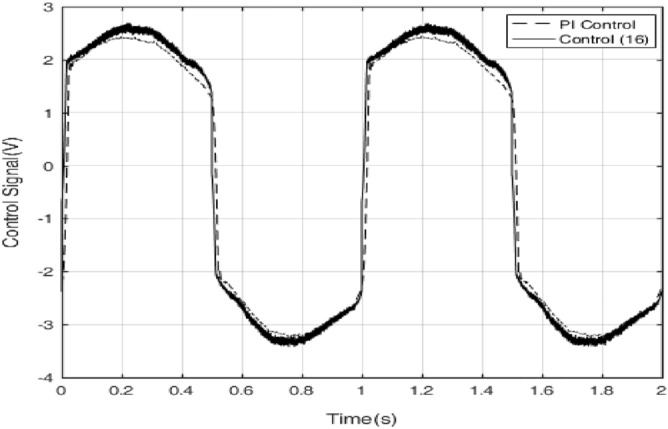
Comparison of the control signals of PI controller and control method (16) with 0.3 kg load.

**Figure 14:**
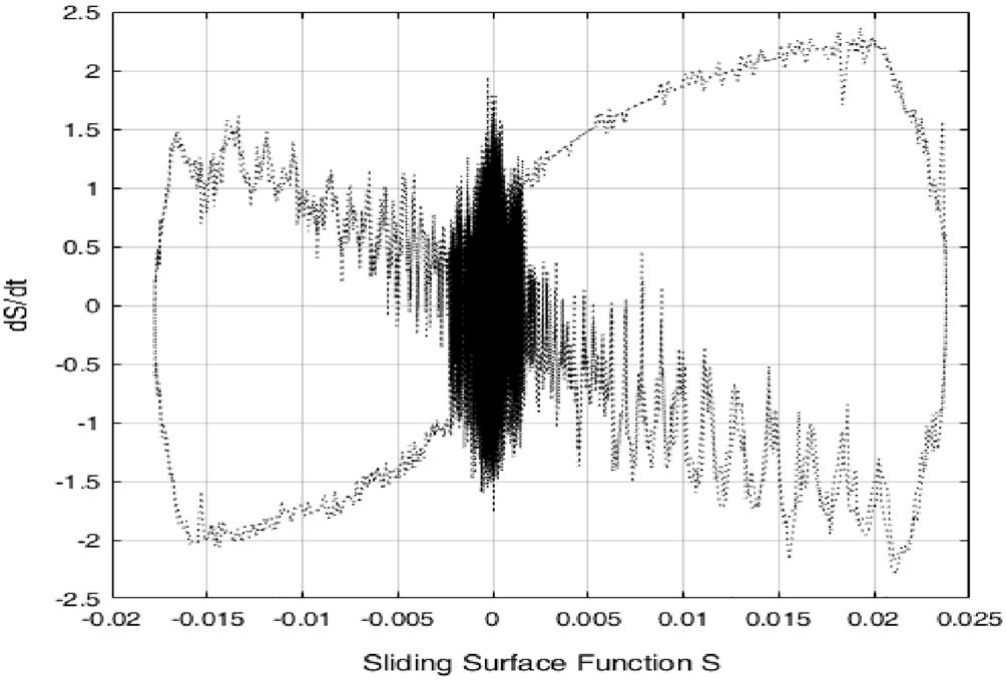
$s$ versus $\dot{s}$ in the phase plane with 0.3 kg load.

Finally, in order to illustrate the robustness of the approach of PID operation of the sliding surface plus the inverse system compensation, an extra load of 0.3 kg is added to the ultrasonic motor control system, the control parameters remain unchanged. Figure 12 shows the errors with extra load. The control signals with extra load of the control method (16) and PI control are shown in Fig. 13. The sliding surface function $s$ and its derivative $\dot{s}$ with extra load are shown in Fig. 14. It can be seen that $s$ and $\dot{s}$ are still well convergent in the phase plane.

## Conclusions

In this paper, a high precision tracking control approach of the ultrasonic motor system is given by using the approach of PID operation of sliding surface approach with inverse system feed-forward compensation, by observing Fig. 9 and Fig. 12, the control method proposed in this paper has no switching action, which avoids the generation of chattering and improves the performance of system. It also can be found that the velocity at zero-crossing will produce large error, which is due to the influence of static friction force. If the static friction force can be compensated in time, the control precision is expected to be further improved. In addition, the higher the precision of the model is, the smaller the error after the inverse system compensation will be, the needed compensation of the sliding mode control will be more smaller, the performance of the system will be higher. The method presented in this paper has strong generality. Further work is how to improve the accuracy of the model, the focus is how to effectively compensate for static friction, which will further improve the tracking accuracy of control system.
